# How does a Truss conservative endodontic access cavity impact the degree of intra-canal disinfection and postoperative pain?

**DOI:** 10.1038/s41432-026-01212-4

**Published:** 2026-03-09

**Authors:** Christopher Rae, David Edwards, David Lynch

**Affiliations:** Clinical Fellow in Restorative Dentistry, School of Dental Sciences, Newcastle University, Framlington Place, Newcastle upon Tyne, UK

**Keywords:** Dental diseases, Dental pulp

## Abstract

**A Commentary on:**

**Özen MM, Karataş E**.

Effect of Cavity Design on Bacterial Reduction in Root Canals and Postoperative Pain Level: A Randomised Controlled Trial. *Aust Endod J.* 2025. 10.1111/aej.12976.

**Study design:**

A randomised controlled trial evaluated the impact of endodontic access cavity design on intra-canal bacterial reduction and postoperative pain. Patients were allocated to either the Traditional Conservative Endodontic Cavity (TEC) or Truss Endodontic Cavity (TREC) group using computer randomisation. Microbial samples were collected from the mesial root canals of lower molars at baseline and following chemo-mechanical preparation. The total number of bacteria present and the percentage reduction following instrumentation were analysed via real-time quantitative polymerase chain reaction (RT-qPCR). Samples were evaluated for bacterial load using 16S rRNA universal primers. Postoperative pain was recorded daily for 7-days using a Visual Analogue Scale (VAS).

**Case selection:**

250 patients attended for assessment at the Endodontics Clinic of A.U. Faculty of Dentistry, Turkey. 100 patients (*n* = 50 per group) met the inclusion criteria (mainly including necrotic mandibular molars with asymptomatic apical periodontitis, where there has been complete root development), 96 patients completed the study (*n* = 48 per group).

**Data analysis:**

Sample size calculation to achieve 95% power and 5% alpha error identified a minimum of 17 participants were required to evaluate antibacterial efficacy, with 27 participants required to evaluate postoperative pain. The statistical power was enhanced by sampling 50 patients in each of the observed groups. Blinding of operators was not possible, and information about individuals conducting the analysis was not included. Statistical analysis was performed using SPSS v20.0. Categorical variables were analysed using the Chi-square test. Normality and homogeneity of numerical data were evaluated using the Kolmogorov-Smirnov and Levene’s tests, respectively. Given the non-normal distribution of the data, intergroup comparisons of age, bacterial load, and post-operative pain levels were conducted using the Mann–Whiteny U test. To compare bacterial load and postoperative pain levels within the groups, the Wilcoxon test and Friedman test were applied, respectively. Statistical evaluations were carried out at the 95% confidence interval.

**Results:**

Intra-canal bacterial count was significantly reduced in both groups following chemo-mechanical preparation. There was a bacterial load reduction from 6.4 × 10^5^ to 4.8 × 10^4^ (96.32%) in the TEC group and a reduction from 6.7 × 10^5^ to 5.2 × 10^4^ (92.32%) in the TREC group. This difference in the percentage of total bacterial count reduction was statistically significant between the groups (*p* > 0.05). Therefore, the null hypothesis can be rejected at the 95% confidence interval. For postoperative pain, both groups demonstrated a statistically significant reduction in postoperative pain levels from day 1 to day 7. There was no statistically significant difference in pain levels between the two groups at any time point.

**Conclusions:**

Endodontic access cavity design impacts reduction in bacterial load following chemo-mechanical preparation. The TEC allowed for greater reduction in bacterial levels compared to the TREC. This is likely due to reduced access restricting effective debridement. There are benefits to retaining more dentine with regards to improved fracture resistance following treatment, so further research is required in this area. Additionally, neither the TEC nor TREC had any impact on postoperative pain levels.

## GRADE Rating:



## Commentary

Endodontic access cavity design is critical for successful root canal treatment, enabling canal location, disinfection, instrumentation and obturation whilst minimising procedural mistakes such as perforation or ledging. There has been a clear shift to providing more minimally invasive treatment across the profession, to preserve sound hard tissue wherever possible. Techniques such as the TREC have gained popularity due to their minimally invasive nature and obvious benefits with regards to post-endodontic treatment restoration^1^.

The Truss Endodontic Access Cavity aims to preserve residual tooth structure, which involves directly accessing from the occlusal surface to expose the mesial and distal canal orifices and keep the intervening dentine intact^2^. There will be a theoretical increased fracture resistance, potentially lessening the need for complex cuspal coverage restorations. However, a recent systematic review highlights the limited benefit of TREC design over conventional TEC in terms of resistance to fracture^1^. In addition, the TREC presents notable challenges, as the technique requires additional postgraduate training, use of magnification, and ultra flexible file systems. If not conducted carefully, poorer disinfection and procedural mistakes such as ledging or perforation may occur.

Given these challenges, and potential lack of benefit in terms of fracture resistance, justification is needed for their use. This study investigated the bacterial load reduction and postoperative pain following endodontic treatment using either Traditional Endodontic access cavity (TEC) or a Truss Endodontic access cavity (TREC). Ethical approval was obtained from the local ethics committee, and the trial was supported by the Scientific Research Projects Coordination unit of A.U.

Although the study was conducted in an endodontic clinic, the qualification level of those assessing the patients and providing the treatment/sampling was not stated.

Investigators could not be blinded to the intervention, and details regarding blinding during outcome analysis were absent. Informed consent was obtained from participants, although there is no confirmation of whether they were blinded to the technique used. This leaves the paper open to the potential of bias.

Sample size was determined using G*Power 3.1 software based on studies with large effect sizes. Investigators increased the sample size beyond the recommended numbers which increased the statistical power. Homogeneity of the samples between groups appeared sound. There was a strict inclusion and exclusion criteria.

Treatment was carried out in the same manner other than the studied intervention, so can be reliably compared. The microbial samples were gathered by inserting sterile paper points “as deeply as possible into the canals”. There is potential for inconsistencies in the placement of these paper points, and therefore sampling as a result.

A superior bacterial load reduction (96%) was recorded in the TEC group. This is thought to be due to increased access leading to better debridement of the entire root canal system. Although the comparative literature presents mixed results, there is a clear pattern that effective mechanical instrumentation can be compromised by cavity design and may affect the elimination of bacteria^3^.

Activation of irrigant solutions provides significantly higher biofilm reduction than conventional irrigation methods^4^, and as this was not included in the workflow of the procedures, may have contributed to the percentage bacterial load reduction in each group. The existing literature suggests that TREC will be more limiting as the presence of the pulp chamber roof will prevent adequate access of activating instruments^5^.

There was no statistically significant difference between the groups when investigating postoperative pain. These findings are consistent with existing research, although this area is limited. It is thought that if the treatment protocols are the same, then there will be an insignificant difference in extruded apical material, and resultant postoperative pain.

General dental practitioners face limitations in providing minimally invasive access cavities, often due to time constraints or experience. While the use of the TREC may offer superior retention of hard tissue, its implications for long-term outcomes require broader consideration. Factors such as canal disinfection, risk of missed canals, and procedural errors; including ledging, instrument fracture, and perforation, must be weighed against the benefits of conservative access. These risks are strongly influenced by operator skill level and the availability of adjunctive technologies such as magnification, illumination, CBCT, ultrasonics, and flexible file systems, which are not universally accessible in general practice^6^.

While the TREC may improve fracture resistance, its added complexity, insignificant impact on postoperative pain and reduction in disinfection are key limitations. Restoration planning is critical with any endodontic treatment; if cuspal coverage is indicated, the impact of access design on fracture resistance may be clinically negligible. Furthermore, the study’s restriction of the inclusion criteria (most specifically to mandibular molars) limits application to premolars or heavily restored teeth, where structural compromise and canal complexity differ significantly. For general practitioners, the priority remains achieving predictable disinfection and canal location while minimising iatrogenic errors.

Future research should explore these variables across diverse tooth types and restorative scenarios to establish evidence-based guidelines that balance bacterial control, structural integrity, and practical feasibility.

Practice points

- TREC access cavity design may hinder effective debridement of root canals.
- Appropriate case selection is essential. Factors such as fracture resistance, risk of missed canals, and procedural errors must be weighed against the benefits of conservative access.
- Successful application of minimally invasive access cavity design is strongly influenced by operator skill and the use of adjunctive technologies, which may not be available in general practice.
- There is no significant difference in postoperative pain between TREC or TEC access cavity design^7^.
